# Duplication of the *EFNB1* Gene in Familial Hypertelorism: Imbalance in Ephrin-B1 Expression and Abnormal Phenotypes in Humans and Mice

**DOI:** 10.1002/humu.21521

**Published:** 2011-05-03

**Authors:** Christian Babbs, Helen S Stewart, Louise J Williams, Lyndsey Connell, Anne Goriely, Stephen RF Twigg, Kim Smith, Tracy Lester, Andrew OM Wilkie

**Affiliations:** 1 Weatherall Institute of Molecular Medicine, University of Oxford, John Radcliffe HospitalOxford, United Kingdom; 2 Department of Clinical Genetics, Oxford Radcliffe Hospitals NHS Trust, Churchill HospitalOxford, United Kingdom; 3 Medical Genetics Laboratories, Oxford Radcliffe Hospitals NHS Trust, Churchill HospitalOxford, United Kingdom

**Keywords:** hypertelorism, Teebi, CFNS, *EFNB1*, cellular interference, imbalance

## Abstract

Familial hypertelorism, characterized by widely spaced eyes, classically shows autosomal dominant inheritance (Teebi type), but some pedigrees are compatible with X-linkage. No mechanism has been described previously, but clinical similarity has been noted to craniofrontonasal syndrome (CFNS), which is caused by mutations in the X-linked *EFNB1* gene. Here we report a family in which females in three generations presented with hypertelorism, but lacked either craniosynostosis or a grooved nasal tip, excluding CFNS. DNA sequencing of *EFNB1* was normal, but further analysis revealed a duplication of 937 kb including *EFNB1* and two flanking genes: *PJA1* and *STARD8*. We found that the X chromosome bearing the duplication produces ∼1.6-fold more *EFNB1* transcript than the normal X chromosome and propose that, in the context of X-inactivation, this difference in expression level of *EFNB1* results in abnormal cell sorting leading to hypertelorism. To support this hypothesis, we provide evidence from a mouse model carrying a targeted human *EFNB1* cDNA, that abnormal cell sorting occurs in the cranial region. Hence, we propose that X-linked cases resembling Teebi hypertelorism may have a similar mechanism to CFNS, and that cellular mosaicism for different levels of ephrin-B1 (as well as simple presence/absence) leads to craniofacial abnormalities. Hum Mutat 32:1–9, 2011. © 2011 Wiley-Liss, Inc.

## Introduction

Ocular hypertelorism (hereafter referred to as hypertelorism), meaning abnormally widely spaced eyes, may manifest in isolation (MIM♯ 145400), as the primary abnormality in brachycephalofrontonasal dysplasia (Teebi hypertelorism) (MIM♯ 145420), or in association with several syndromes. Hypertelorism is prominent in craniofrontonasal syndrome (CFNS; MIM♯ 304110) and is the only feature of this X-linked developmental disorder that is commonly shared by heterozygous females and hemizygous males [Grutzner and Gorlin, 1988]. Females with CFNS are paradoxically more severely affected than males, additionally exhibiting frontonasal dysplasia with a central nasal groove, unilateral or bilateral coronal craniosynostosis, and extracranial features, whereas males normally have no other malformations. The classical CFNS female phenotype is caused by heterozygous loss-of-function mutations in *EFNB1* (MIM♯ 300035), which encodes the transmembrane signaling molecule ephrin-B1 [Twigg et al., 2004; Wieland et al., 2004]. Over 80 distinct mutations of *EFNB1* have been reported in CFNS; these molecular lesions show a wide diversity ranging from missense substitutions to complete gene deletions, but no genotype–phenotype correlation is evident. All CFNS mutations are believed to cause partial or complete loss of ephrin-B1 function [Twigg and Wilkie, 2008].

Ephrins and their Eph receptors have been shown to play important roles in development and homeostasis through control of several cellular processes mediated via bidirectional cell contact-dependent signalling [Arvanitis and Davy, 2008; Pasquale, 2008]. Frequently, expression of Eph receptors and ephrins is reciprocal and, through repulsive interactions at interfaces, organizes positioning and/or movement of cells including the restriction of migrating neural crest cells (NCC) to specific embryonic territories [Wilkinson, 2001]. Murine *Efnb1* is expressed in neural crest but not in the cephalic mesoderm; hence, the coronal suture, which develops at the interface between these tissues, lies at the boundary of *Efnb1* expression [Twigg et al., 2004]. The occurrence of craniosynostosis in CFNS suggests that ephrin-B1 may play a role in establishing and/or maintaining the separation of these tissues.

Insight into the paradoxical inheritance pattern of CFNS was provided by analysis of the knock-out (KO) *Efnb1* mouse, in which heterozygous females also have a more severe phenotype than either hemizygous males or homozygous females [Compagni et al., 2003]. This has led to a model of pathogenesis whereby the process of random X-inactivation in the heterozygous female leads to patchy ephrin-B1 expression, which is accentuated by the homophilic sorting of cells that do, or do not, express ephrin-B1. The result is that ectopic tissue boundaries are formed, a process that has been termed “cellular interference,” and cannot occur in hemizygous males [Wieland et al., 2004]. The milder phenotypes observed in males suggest that ephrin-B1 function is redundant in most tissues where it is expressed. Further indications that ephrins play a wider role in the developing skull vault come from the identification of heterozygous mutations in *EFNA4*, encoding ephrin-A4, in patients with nonsyndromic unicoronal craniosynostosis [Merrill et al., 2006], and from partial fusion of the coronal sutures in mice homozygous for an *EphA4* null allele [Ting et al., 2009].

Here we report a female patient with severe hypertelorism and mild nail ridging but without craniosynostosis or other features of CFNS. Because of the phenotypic overlap with CFNS, the patient's DNA was screened for mutations and for copy number variation in *EFNB1* by sequence analysis and multiplex ligation-dependent probe amplification (MLPA), respectively. Unexpectedly, MLPA revealed a duplication of all five exons of *EFNB1*. We present the further molecular genetic investigation of this family and show that the duplicated *EFNB1* allele generates approximately 1.6-fold as much transcript as the normal allele. We also provide evidence that allelic imbalance in a mouse model (*Efnb1*^Lox^) targeted to contain a human *EFNB1* cDNA in place of the endogenous *Efnb1* gene results in aberrant cell mixing of the cranial primordia during development. Taken together, these data suggest that cellular mosaicism for different levels of ephrin-B1 (as well as simple presence/absence) leads to craniofacial abnormalities.

## Methods

### Clinical Ascertainment and Initial Diagnostic Studies

The family was initially referred to the local clinical genetics service for assessment. Ethical approval for research into craniofacial malformations was provided by the Oxfordshire Research Ethics Committee B (C02.143), and informed consent was obtained from all participants by clinical staff, in accordance with local guidelines. Genetic analyses were performed on DNA and RNA extracted from peripheral blood. The human genome hg18 sequence release (March 2006) was used for all analyses presented in this article.

The duplication was originally detected by MLPA analysis conducted according to the manufacturer's instructions using the P080 kit (MRC Holland). The extent of the duplication was determined by array comparative genomic hybridization (array CGH) using a 244 K array (Agilent Technologies, Englewood, CO) according to the manufacturer's instructions. The hybridized arrays were washed and scanned using an Agilent Microarray Scanner, the image data were extracted using Agilent Feature Extraction software version 8.5, and analyzed using Agilent CGH Analytics software version 3.4 (*z*-score method setting). Fluorescence in situ hybridization (FISH) characterization of the duplication was conducted using a bacterial artificial chromosome (BAC) clone RP11-30P7 (Invitrogen, Carlsbad, CA) that spans the *EFNB1* gene region. Probe generated from BAC clones was labeled with digoxygenin and detected with fluorescein.

### Isolation of Duplication Breakpoint

Single copy probes spanning a 36-kb region to include the centromeric limit of the duplicated segment were synthesized and hybridized to Southern blots of patient and control DNA. Initially, a 6-kb *Hind*III breakpoint fragment was identified that indicated the breakpoint lay in a 1,933-bp region. Further localization on Southern blots by looking for additional breakpoint fragments revealed the order and relative positions of restriction enzyme sites around the telomeric limit of the duplicated segment. A restriction map of a 26-kb region containing this telomeric limit was generated from published sequence and compared to the map determined from Southern analysis. This allowed the breakpoint to be identified as lying within a ∼1-kb region. A primer pair was designed that amplified a unique 1,095-bp product from the patient's DNA: 5′-TGCATCGCTCATGCTGGGAGCTGTAGAC-3′ and 5′-GTGCTGTATTCAGGAGACCCATATCACGTGCA-3′. After DNA sequencing to determine the site of the breakpoint, a further primer pair (5′-GGTAATGTGTCATTTCCCTCTGTCTCCTTTCAG-3′ and 5′-ACAGAAACTGAACGACCTGCTCTTGAATGACTA-3′) was designed to confirm the breakpoint in other family members by amplifying a 250-bp fragment in a multiplex reaction with a normal control primer pair designed to amplify a fragment of *GLI2* on chromosome 2 (5′-TGTCGGGGACATGAGCTCCATGCTCAC-3′ and 5′-CCGGAGCAGAGTATCCAGTATAGCATTCAGA-3′).

### Relative *EFNB1* Expression and Correction for Skewed X-Inactivation

**Figure 4:**
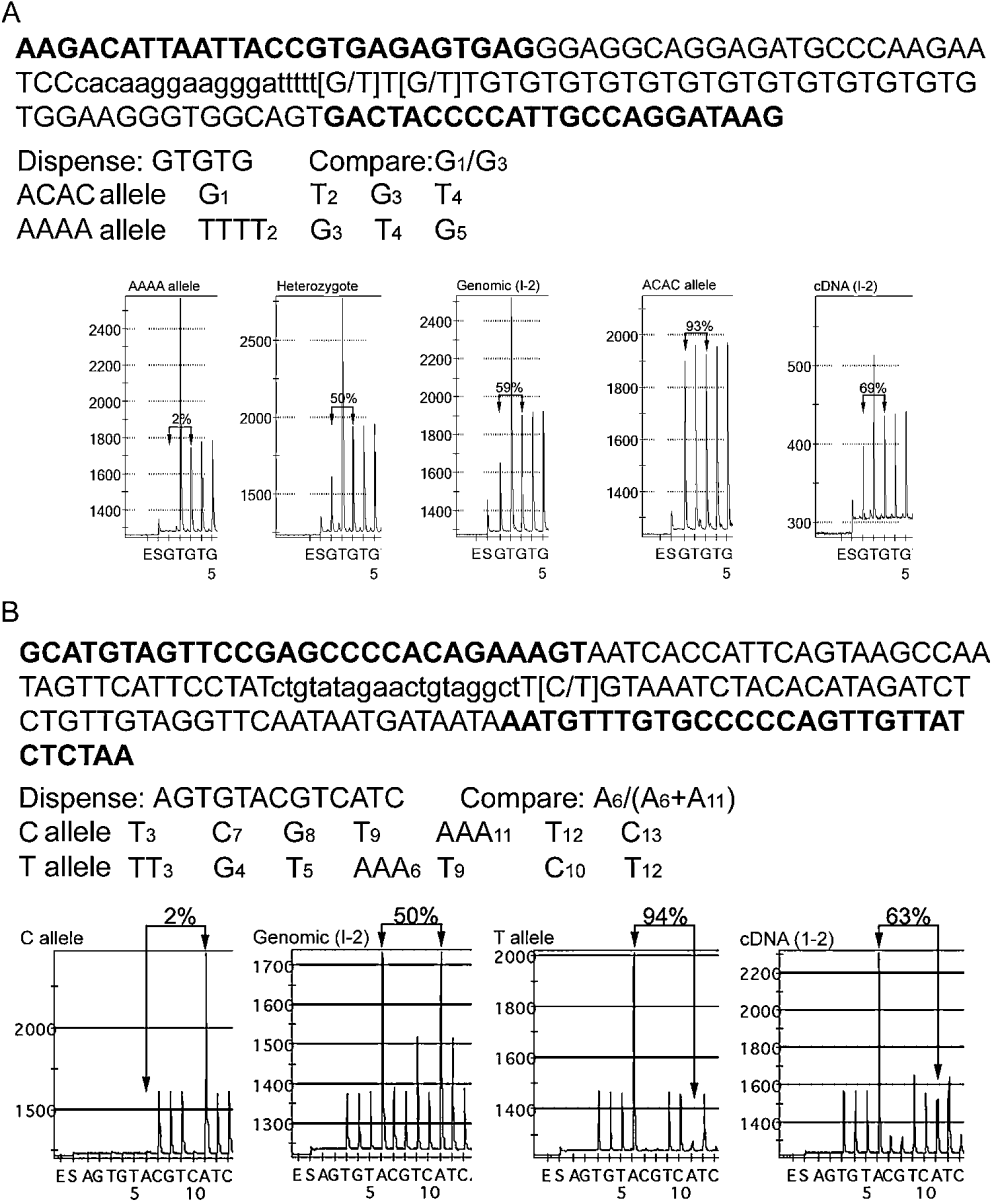
Quantification of relative allelic transcription at *EFNB1* (**A**) and *XIST* (**B**) by Pyrosequencing. In each figure, the part of the cDNA sequence analysed is shown at the top, with the amplification primer sequences in bold, sequencing primers in lower case, and the polymorphic base(s) enclosed by square brackets. Below, the dispensation order and allele-specific bases for comparison are indicated, with a table listing the allele-specific incorporation of bases at each dispensation (denoted by subscripted numbers). The lower part shows pyrograms obtained using genomic DNA from hemizygous males carrying each of the alleles under investigation, genomic DNA from subject I-2, genomic DNA from a normal female and cDNA generated from peripheral blood from subject I-2. The allele-specific peaks used for quantification are marked with arrows and raw measurements of comparative ratios indicated as percentages.

Total RNA was isolated from peripheral blood using the PAXgene Blood RNA Kit (Qiagen, UK), treated with DNAseI (Roche, Indianapolis, IN), and cDNA was generated using random hexamer primers (RevertAid, Fermentas, Hanover, MD). cDNA was amplified using primers shown in Figure 4 and the SNPs rs16990748 and rs688969, which are in complete linkage disequlibrium and lie 2 bp apart in the 3′ untranslated region of *EFNB1*, were used to quantify the relative expression of the *EFNB1* alleles in the heterozygous individual I-2. Correction for differential X-inactivation was achieved using the SNP rs1620574 in *XIST*, which was also heterozygous in I-2.

Relative amounts of each allele were quantified by pyrosequencing. Single-stranded PCR products (see Fig. 4 for details) were obtained by using a single biotinylated primer, immobilization on streptavidin beads, and denaturation with NaOH. Products were annealed to the sequencing primer by heating to 80°C for 2 min. Pyrosequencing [Langaee and Ronaghi, 2005] was performed on three independent amplification products from control and patient samples on a PSQ-HS96A system (Qiagen), with use of primers and dispensation orders designed to quantify the normal and duplicated allele of *EFNB1* and *XIST* alleles. Each pyrogram was required to pass three criteria to ensure only high-quality data were analyzed: absence of duplication-specific peaks in control samples, no decrease in peak height as dispensation progressed, and no unexpected peaks from blank dispensations. Peak heights were recorded using PSQ96 SQA software and the values exported to a MS Excel data sheet. For each pair of peaks selected for quantification, the normal/duplicated peak ratio was obtained. Quadratic interpolation was applied for deviations from expected 0, 50, 67, and 100% for the control samples.

The correct phase between the duplicated *EFNB1* allele and *XIST* SNP in subject I-2 was confirmed by amplifying 10 microsatellites in the four siblings of I-2 and in the three affected family members, using primers labelled with the fluorophore 6-FAM (primer pairs are shown in Supp. Table S1). Fragments were analysed by capillary electrophoresis on an ABI 3130 containing POP-7 polymer. Peaks were visualized using Gene Mapper v3.7 (Applied Biosystems, Bedford, MA).

### Analysis of *Efnb1*^Lox^ Mice

Mice carrying a conditional *Efnb1*^Lox^ allele on a C57Bl/6J background [Compagni et al., 2003] were a gift from R. Adams (London Research Institute, UK). X-inactivation in embryos was analyzed using mice carrying an X-linked green fluorescent protein (*GFP*) transgene (D4/XEGFP) [Hadjantonakis et al., 2001]. EphB2 expression was determined by β-galactosidase staining in mice carrying an allele targeted to generate an EphB2–LacZ fusion protein [Henkemeyer et al., 1996].

For β-galactosidase staining, embryos were fixed in 1% formaldehyde, 0.2% glutaraldehyde, 5 mM EGTA, 2 mM MgCl_2_, and 0.02% NP-40, and incubated in staining solution containing 5 mM potassium ferricyanide, 5 mM potassium ferrocyanide, 2 mM MgCl_2_, 0.01% sodium deoxycholate, 0.2% NP-40, and 1 mg/mL X-gal.

To visualize GFP on histological sections, embryos were fixed in 4% paraformaldehyde at 4°C, washed three times in phosphate-buffered saline, embedded in OCT-compound (R.A. Lamb), and cut on a cryostat (Leica, Deerfiled, IL) at 15 µM. Specimens were viewed and photographed using a Leica MZFLIII microscope and DFC300F camera. Images were viewed and stored using Openlab software (Improvision).

Specimens for micro-CT were scanned using a General Electric Locus SP micro-CT scanner (GE Healthcare, Piscataway, NJ). The specimens were immobilized using cotton gauze and scanned to produce 14–28 µm voxel size volumes. The specimens were characterized further by making three-dimensional isosurfaces, generated and measured using Microview software (GE Healthcare).

Expression of the *EFNB1* cDNA was compared with endogenous *Efnb1* in *Efnb1*^Lox^ and wild-type (wt) male mice, respectively. Total RNA was isolated from adult brain and E15.5 embryonic heads using RNA-Bee (AMS Biotechnology, Abingdon, UK), treated with DNAseI (Roche), and cDNA was generated using random hexamer primers (RevertAid, Fermentas). Quantification of expression by real-time PCR was conducted using a primer pair to amplify a cDNA fragment spanning exon 1 and exon 2 of the endogenous *Efnb1* gene that is preserved in the *Efnb1*^Lox^ targeted allele (5′-CAGGCGAGGCGAGCTTTGCTGAG-3′ and 5′-CAGCTTGTCTCCAATCTTCGGGTAGATCAC-3′). Expression was normalized using two control genes, *Gapdh* (5′-CATGGCCTTCCGTGTTCCTA-3′ and 5′-CCTGCTTCACCACCTTCTTGAT-3′) and the ribosomal protein encoding gene *Rplp0* (5′-CACTGAGATTCGGGATATGCTGTTGGCCAAT-3′ and 5′-CTTCTCGGGTCCTAGACCAGTGTTCTGA-3′). Amplification reactions were monitored using SYBR Green I Master (Roche) in a Light Cycler 480II (Roche). Between six and eight independent reactions were obtained for each tissue/primer pair combination. Results are expressed as the mean ± standard error of the mean (SEM) of the cycle at which each reaction reached a predetermined threshold (Ct) relative to the wt level.

## Results

### Case Report

**Figure 1:**
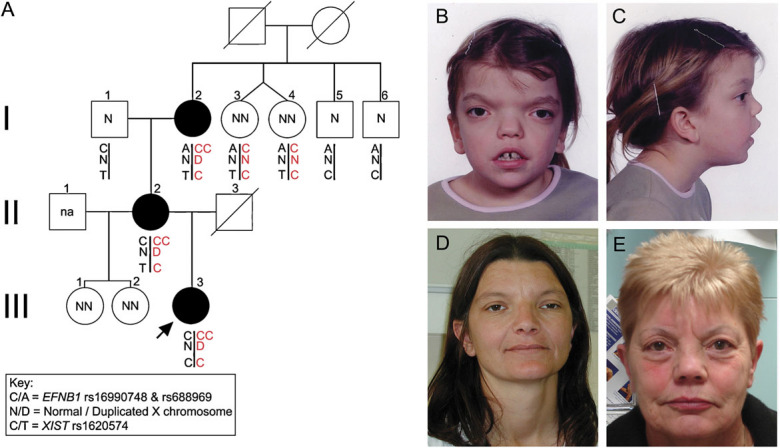
Pedigree and phenotype of individuals heterozygous for *EFNB1* duplication. **A:** Pedigree showing the immediate family of the proband (arrow). Filled symbols represent individuals shown to carry the duplication. Under each symbol, haplotypes of loci assayed in *EFNB1* and *XIST* are shown together with duplication status of each X chromosome (see key). Note that the X chromosome bearing the duplication (marked in red in the duplicated individuals and in the nonduplicated sisters of I-2) is identified by a C allele at the *EFNB1* and *XIST* SNPs. na indicates that no DNA was available for analysis. **B,C**: Facial appearance of the proband, aged 10 years. **D:** Proband's mother (II-2) aged 41 years and E, grandmother (I-2) aged 63 years.

The proband III-3 (pedigree shown in Fig. 1A) was referred for a clinical genetics opinion at the age of 7.9 years because of dysmorphic features, short stature, and mild developmental delay. On examination she was noted to have marked hypertelorism, a widow's peak, frontal bossing, thick eyebrows, a short nose, and arched upper lip (Fig. 1B and C). There was mild nail ridging. She attended a normal school but required additional support in class. She had been born at 38 weeks' gestation with a weight of 3.55 kg (+1.3 SD), walked at 18 months, and had no words until 2 years. Formal measurements at the age of 9.0 years documented a height of 111 cm (−3.8 SD), head circumference 55 cm (+2.4 SD) and inner canthal distance 4.4 cm (+6 SD). An umbilical hernia required surgical repair at the age of 11 years. In the family history, hypertelorism was present in her mother II-2 (Fig. 1D) (inner canthal distance 4.2 cm, +4.7 SD), who exhibited mild nail ridging, and maternal grandmother I-2 (Fig. 1E) (inner canthal distance 4.0 cm, +3.6 SD); both were generally healthy and of normal stature (173 cm [+1.6 SD] and 165 cm [+0.2 SD], respectively) and intelligence and neither had a history of recurrent miscarriages. No other family members had hypertelorism. Three-dimensional computed skull tomography of subject III-3 demonstrated hypertelorism with vertical displacement of the right orbit. There was no evidence of craniosynostosis and the appearance of the brain was normal. The remainder of the skeletal survey was normal.

### Characterization of a 937-kb Duplication Including *EFNB1*

**Figure 2:**
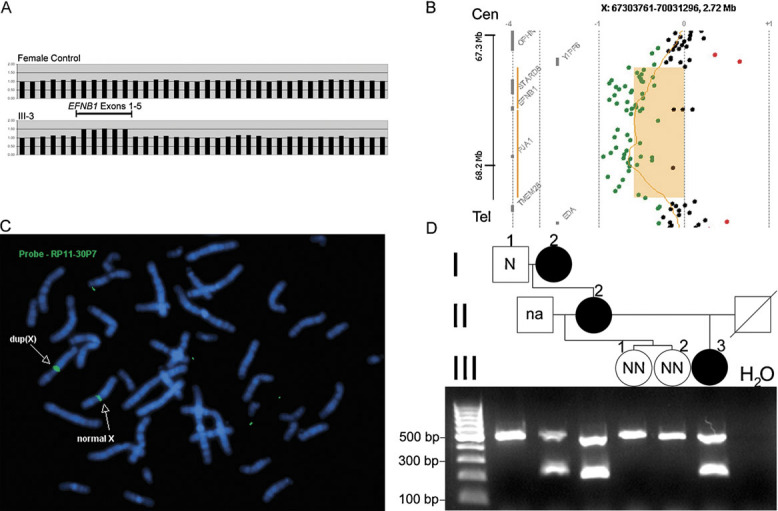
Molecular and cytogenetic characterization of X-chromosome duplication including *EFNB1*. **A:** MLPA analysis of the proband III-3 (lower panel) compared with a normal female control (upper panel). The estimated dose of *EFNB1* exons 1–5 in the proband is ∼1.5 compared with 31 autosomal amplicons. **B:** Plot showing extent of duplication determined by array CGH. Probes apparently duplicated in the proband are shown in green, indicating a duplication size of ∼900 kb. **C:** FISH with BAC clone RP11-30P7. This clone spans the *EFNB1* gene and hybridizes to the X chromosomes only, with one chromosome showing a brighter signal, consistent with a tandem duplication of the *EFNB1* region. **D:** Duplex PCR assay for duplication junction (lower fragment) and *GLI2* amplification control (upper fragment). Pedigree symbols are aligned above corresponding lanes; H_2_O, water control. na indicates that no DNA was available for analysis.

The segregation of hypertelorism in three generations suggested a possible diagnosis of Teebi hypertelorism syndrome, but in view of the clinical overlap with CFNS, mutation testing of *EFNB1* was arranged. Screening for mutations in the coding region was normal, but MLPA analysis revealed a duplication of all five exons of *EFNB1* (Fig. 2A). Subsequently, array CGH using a 244 K oligonucleotide array (see Methods) confirmed the presence of a duplication within the band Xq13.1, including the *EFNB1* gene, approximately 888–950 kb in size (Fig. 2B). FISH analysis showed that the duplication appeared to have occurred in tandem on Xq13.1 (Fig. 2C).

**Figure 3:**
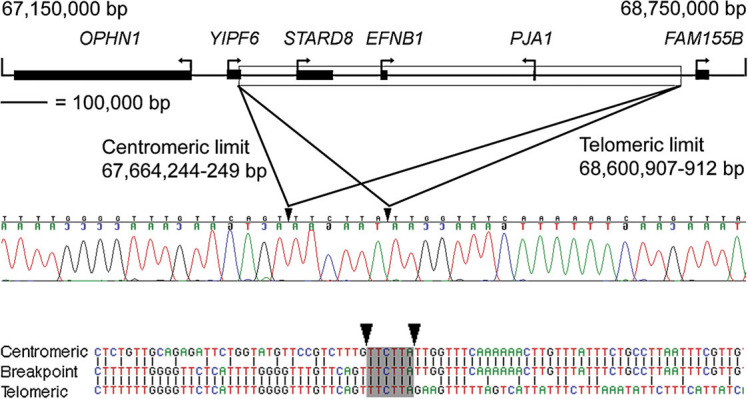
DNA sequence characterization of X chromosome duplication. The upper panel shows positions and directions of transcription of genes in the region and the size and extent of the duplicated segment (rectangular box). Below, the DNA sequence chromatogram spanning the breakpoint and alignment of this sequence compared to the normal sequences at the telomeric and centromeric limits of the duplication. The arrowheads indicate the range of possible positions of the breakpoint within the six-nucleotide identity (shaded) shared by both normal sequences. [Color figures can be viewed in the online issue, which is available at http://www.wiley.com/humanmutation.]

The sequence around the duplication limits, delineated by array CGH (Fig. 2B), was analyzed in order to design single copy probes for Southern blotting. Because the telomeric limit of the duplication contained greater than 95% repetitive sequence, we designed probes to span the centromeric limit. Following two rounds of restriction site mapping by Southern blot analysis, the duplication limits were sufficiently localized to enable design of a PCR primer pair to amplify across the duplication breakpoint (see Methods). DNA sequencing of this product identified the molecular structure of the breakpoint (Fig. 3). The sequences show no significant similarity except for a shared 6-bp TTCTTA motif. The centromeric duplication limit lies between 67,664,244 and 67,664,249 bp and the telomeric duplication limit between 68,600,907 and 68,600,912 bp, revealing the size of the duplicated region as 936,663 bp. Analysis of the normal sequences at the two breakpoints shows the proximal breakpoint to be in an L1 LINE (L1P2) repetitive element, whereas the distal breakpoint resides in the 3′ terminal end of an L1MDa repetitive element.

We designed a duplex PCR assay to simultaneously amplify the duplication breakpoint and a control sequence in *GLI2* (see Methods) to determine whether other family members carried the duplication. We found the identical duplication in the proband's mother II-2 and maternal grandmother I-2 (Fig. 2D). The duplication was not present in the proband's half sisters III-1 and III-2, her maternal grandfather I-1 (Fig. 2D), or in any of the four siblings of I-2 (not shown). These results were confirmed by MLPA. The MLPA analysis of the sample from the blood of I-2 indicated a 1.5-fold increase in dosage of all five exons of *EFNB1*, suggesting a complete rather than mosaic duplication in this individual (not shown).

The duplication includes three complete genes (*STARD8*, *EFNB1*, and *PJA1*), while the final exon of *YIPF6*, a 7-exon gene, is also duplicated. We found exact segregation of the 937-kb duplication with the hypertelorism phenotype in the family (Fig. 1A). Given the similarity of the phenotype in this family to CFNS, we speculate that the duplication of the *EFNB1* gene is likely to cause the hypertelorism. We therefore sought to determine whether the X-chromosome that carries the duplication produces a larger amount of *EFNB1* mRNA.

### Quantification of *EFNB1* Expression Level and Correction for Skewing of X-Inactivation

To develop an assay for imbalance in *EFNB1* mRNA expression, we searched for an informative polymorphism by sequencing the entirety of *EFNB1*, including the 5′ and 3′ untranslated regions (UTRs) in the three affected family members. II-2 and III-3 were uninformative, but I-2 was heterozygous for two closely adjacent A/C polymorphisms (rs16990748 and rs688969). This enabled us to distinguish in subject I-2 the X chromosomes carrying the normal (AAAA genotype) and duplicated (ACAC genotype) copies of *EFNB1* (Fig. 1A). A Pyrosequencing assay was designed (see Methods and Fig. 4A) to quantify the relative expression of the two alleles in cDNA generated from peripheral blood. From this we estimated that 74.0% (SEM 2.3%; *n* = 3) of the *EFNB1* expressed in these cells originated from the duplicated allele (Fig. 4A).

To correct for any skewing of X-inactivation, we screened the DNA of subject I-2 for linked informative polymorphisms. The well-characterized trinucleotide repeat within the *AR* locus was not informative, but subject I-2 was heterozygous for a C/T polymorphism (rs1620574) within the *XIST* locus at 72,960,792 bp, ∼4.36 Mb distal of the telomeric limit of the duplication (Fig. 1A). *XIST* is transcribed exclusively from the inactive X chromosome [Brown et al., 1991] and we adapted a published method [Rupert et al., 1995] to quantify the proportion of each allele in the cDNA samples used to quantify *EFNB1* expression (see Methods and Fig. 4B). Analysis of available family members showed that the C allele was present on the duplicated X chromosome in subjects II-2 and III-3 (Fig. 1A). From the Pyrosequencing assay we estimated that the C allele accounted for 35.8% (SEM 0.3%; *n* = 3) of the total *XIST* transcript level, indicating that the X chromosome containing the duplication was active in 64.2% of cells. Combining these data using the formula (74.0/64.2)/(26.0/35.8) indicates a 1.6-fold increase of *EFNB1* transcription from the duplicated allele.

The genetic distance between *EFNB1* and *XIST* indicates a ∼3.1% chance that a recombination could have occurred between subjects I-2 and II-2, which would reverse the phase of the SNP alleles in I-2. To exclude this, we genotyped 10 informative microsatellite markers in each affected family member and four siblings of I-2 (Fig. 1A). Markers were selected to be either centromeric of *EFNB1* or telomeric of *XIST*, marker locations are shown in Supp. Table S1. Two sisters of I-2 (I-3 and I-4) had identical genotypes to I-2 at all 10 locations, yet did not carry the *EFNB1* duplication; the three sisters shared a haplotype that was transmitted to II-2 and III-3 (data not shown). This makes it very unlikely that a recombination between *EFNB1* and *XIST* has occurred in any of these family members, and also demonstrates that the duplication arose de novo in I-2.

### Abnormal Tissue Mixing in *Efnb1*^Lox/+^ Mice

To investigate further whether imbalance in expression of ephrin-B1 in different cells (as well as simple presence or absence) can lead to phenotypic abnormalities, we obtained mice carrying a *lox*P-flanked human *EFNB1* cDNA cassette knocked into the endogenous *Efnb1* gene on the X-chromosome (here referred to as the conditional *Efnb1*^Lox^ allele) [Compagni et al., 2003]. Mice heterozygous, homozygous or hemizygous for this conditional *Efnb1*^Lox^ allele show reduced viability, ∼10% dying within 24 hr after birth due to cleft palate [Compagni et al., 2003].

The amount of protein produced from the conditional allele was previously reported to be reduced compared with the wt allele, based on Western blotting [Compagni et al., 2003]. However, as the conditional allele contains a human cDNA and the antibody may not bind both mouse and human proteins with equivalent affinity, we undertook to quantify transcript levels. To circumvent the differences between the wt and conditional alleles, a primer pair was designed to amplify a region including exon 1 and exon 2 from the endogenous *Efnb1* gene that is preserved in the conditional allele (see Methods). Expression levels in each tissue were normalized using the transcripts *Gapdh* and *Rplp0*, fragments of which were amplified using previously described primers [Tsujita et al., 2006; Xu et al., 2010]. In adult brain (Supp. Fig. S1), expression of the conditional allele was between 1.69- and 2.75-fold higher than the wt locus. By contrast, in the developing heads of embryonic day (E)15.5 embryos we found no clear difference in expression of the alleles; the conditional allele appeared either increased 1.16-fold or decreased to 0.76-fold according to whether the *Rplp0* or *Gapdh* transcript was used for normalization, respectively. Taken together, these data indicate that expression of the conditional allele varies relative to the wt allele in a time- and tissue-specific manner, and demonstrates significant upregulation in adult brain.

**Figure 5:**
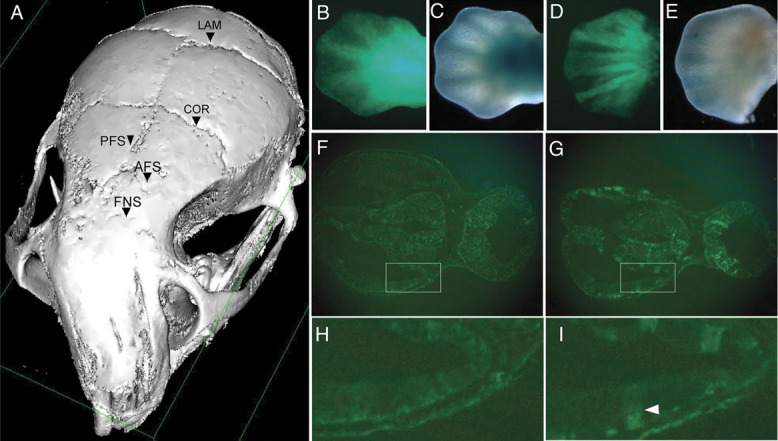
Analysis of *Efnb1*^Lox/+^ mice. **A:** 3D Micro-CT scan of an adult *Efnb1*^Lox/+^ C57BL6/J skull. **B–E:** E12.5 limb buds: **B:** GFP in wt; **C:** EphB2 expression (β-galactosidase staining) in wt. **D:** GFP in *Efnb1*^Lox/+^; E, EphB2 expression in *Efnb1*^Lox/+^. **F,G:** Transverse section through E12.5 heads showing GFP in wt (**F**) and *Efnb1*^Lox/+^ (**G**). **H,I:** higher magnification of areas in rectangles in **F** and **G**, respectively (white arrowhead shows abnormal boundary). Abbreviations: LAM, lambdoid suture; COR, coronal suture; FNS, frontonasal suture; PFS, posterior frontal suture; AFS, anterior frontal suture.

Given that these subtle alterations in expression of the conditional allele partly mimic the effect of the human *EFNB1* duplication, we investigated the craniofacial phenotypes of *Efnb1*^Lox/+^ mice in greater detail. We found that ∼10% of viable *Efnb1*^Lox/+^ heterozygous females exhibit obvious twisting of the skull. Micro-CT scanning shows that this appears to originate in the frontal bones around the level of the anterior frontal suture (Fig. 5A). The interorbital distance of *Efnb1*^Lox/+^ mutants was significantly (1.15-fold) larger compared to wt females of the same strain (wt = 4.2 ± 0.07 [SEM] mm, *n* = 5; *Efnb1*^Lox/+^ = 4.8 ± 0.1 mm, *n* = 5). To observe whether cell sorting is disturbed in female embryos heterozygous for the conditional allele, we crossed *Efnb1*^Lox/+^ females with males carrying both a *GFP* transgene on the X chromosome (XEGFP) and an *EphB2LacZ* allele, and examined embryos at E12.5. Examination of the limbs of wt embryos under ultraviolet light showed random sorting of cells positive and negative for GFP (Fig. 5B). By contrast, the limbs of female *Efnb1*^Lox/+^ heterozygous embryos showed clustering of GFP-positive cells (expressing wt *Efnb1*) and GFP-negative cells (expressing the conditional *Efnb1* allele) as previously demonstrated (Fig. 5D) [Compagni et al., 2003]. To determine whether the abnormal cell mixing is of functional consequence, we stained the embryos with β-galactosidase to determine expression of EphB2, a receptor for ephrin-B1. There was normal EphB2 expression in the distal limb of wt mice (Fig. 5C), but patchy expression in the limbs of mutants (Fig. 5E). Expression of EphB2 appeared increased in the areas where the conditional allele of *Efnb1* was expressed (negative for GFP) and decreased where the wt *Efnb1* transcript was active.

To investigate if the altered cell-sorting is also present in the developing skull vault and neural tissue, transverse sections were taken just above the level of the eye (Fig. 5F and G). Tissue in wt embryos appeared to contain randomly mixed GFP-positive and -negative cells (Fig. 5F and H), whereas in the *Efnb1*^Lox/+^ mutant, clusters of cells were visible (Fig. 5G and I) indicating a coarser pattern of X inactivation likely related to homophilic cell sorting.

## Discussion

This work was originally prompted by the molecular genetic investigation of a girl with severe hypertelorism giving an appearance resembling CFNS. Unexpectedly, she was found to have a duplication of the *EFNB1* gene, which was shown to segregate with hypertelorism also present in the mother and maternal grandmother, in the latter of whom we deduced that the duplication is likely to have arisen de novo. The duplication occurred in the proximity of repeat sequences (L1 LINE and L1MDa repetitive elements) with a six-nucleotide homology at the breakpoint, which most likely indicates a nonhomologous end joining mechanism [Gu et al., 2008]. Because the phenotype in the family overlaps with Teebi hypertelorism, we propose that testing for dosage abnormalities in *EFNB1* would be appropriate in individuals presenting with this diagnosis in cases where X-linkage cannot be excluded.

In addition to *EFNB1*, the duplication interval included two other entirely duplicated genes, *PJA1* encoding the RING-finger protein Praja1 and *STARD8*, which encodes a cell junction protein. Praja1 has been shown to interact with ubiquitin conjugating enzymes and has independent ubiquitin ligase activity [Yu et al., 2002]. In mice *Pja1* is expressed in the developing limbs, facial primordial, and central nervous system at E10.5, and has been suggested to regulate the transcriptional function of the developmentally important homeodomain protein Dlx5 [Gray et al., 2004; Sasaki et al., 2002]. In the mouse *Stard8* is widely expressed; however, neither *STARD8* nor *PJA1* has been implicated in congenital malformations in humans or mice. Our functional studies both of the family and of the conditional mouse (discussed below) support a major contribution of mis-regulated ephrin-B1 expression to the phenotype, but we cannot formally exclude a contribution from duplication of either *PJA1* or *STARD8* in the mechanism of the abnormalities seen in the family reported here.

We have shown that the duplicated X chromosome produces ∼1.6 times as much *EFNB1* mRNA as the normal X chromosome. To explore the functional consequences of this we studied female mice heterozygous for a conditional *Efnb1* allele containing a knock-in of the human cDNA sequence. Expression studies in hemizygous males showed that this conditional allele was significantly (1.69–2.75-fold) overexpressed compared to wt in adult brain, but showed less marked imbalance in embryonic heads (Supp. Fig. S1). Variations in amino acid sequence between murine and human ephrin-B1 (in 12 of 271 residues encoded by the human cDNA) might also contribute to functional differences between the wt and conditional alleles. We observed abnormal cell sorting (reflected in the coarser pattern of X-inactivation and regulation of the receptor protein EphB2) in both the limbs and heads of heterozygous *Efnb1*^Lox/+^ mutant mice, which exhibited significant hypertelorism. Collectively, the findings suggest that abnormal cell sorting per se gives rise to hypertelorism, irrespective of whether this is caused by underactive or overactive *EFNB1* alleles.

Abnormal cell sorting is by definition a noncell-autonomous mechanism; this raises a paradox because hypertelorism (albeit of lesser severity) is also present in males hemizygous for *EFNB1* loss of function mutations. Our finding that relatively mild imbalance in *EFNB1* expression between X chromosomes leads to a more severe phenotype than hemizygous loss of function of *EFNB1*, highlights the predominant contribution of noncell autonomous abnormal sorting to hypertelorism. This is supported by the recent report of a male with abnormalities similar to CFNS, including a very wide midface and pronounced hypertelorism, in whom mosaicism for an additional ring X chromosome (composed of a 24-Mb fragment containing the regions Xp11.3 to Xq13.1, including *EFNB1*) was present in 45% of amniocytes and blood leukocytes [Baker et al., 2010]. It is likely that the mosaicism for *EFNB1* contributed significantly to the craniofacial abnormalities in this child. By contrast a male fetus carrying duplication of *EFNB1* alone exhibited diaphragmatic hernia with no craniofacial abnormalities [Srisupundit et al., 2010]. This suggests that although lack or imbalance of ephrin-B1 may lead to hypertelorism, a uniform excess does not adversely affect craniofacial development.

Analysis of *Xenopus* embryos suggests a mechanism for the homophilic cell sorting observed in the female conditional *Efnb1* mutant mouse embryos studied here [Lee et al., 2008]. This work found that overexpression, as well as morpholino-mediated blocking of endogenous *Efnb1* expression, disrupted both the structure and function of tight junctions in the epithelia of early stage *Xenopus* embryos. These findings indicate that the level of *Efnb1* expression is crucial for maintaining appropriate localization of tight junction-associated proteins; therefore, different levels of ephrin-B1 from each active X chromosome may result in differential cell adhesion between the two cell-populations. Recently, ephrin-B1 expression was found to be regulated posttranscriptionally by *miR-124* [Arvanitis et al., 2010], this complex regulatory modulation further emphasizes the importance of ephrin-B1 levels.

As a result of random X-inactivation in females, cells expressing different levels of ephrin-B1 segregate from one another resulting in patches of homophilically sorted cells. We show that expression of EphB2, an important receptor of ephrin-B1, is upregulated in patches coinciding with transcription of the conditional *Efnb1* allele, resulting in abnormal sites of ligand–receptor interaction. Such boundaries may affect cellular communication as ephrin-B1–EphB2 interaction has been suggested to inhibit gap junction formation by downregulating connexin-43 [Davy et al., 2006]. Taken together, these results provide insight into why females heterozygous for loss of function or altered expression of *EFNB1* develop a significantly more severe phenotype than males hemizygous for loss of function mutations.

The mechanisms by which loss of function of *EFNB1* or imbalance in *EFNB1* expression lead to a consistent phenotype of hypertelorism remain unclear. Normal spacing of the orbits depends on the correct development of a number of components of the cranium: enlargement of the neurocranium at the metopic suture, early growth at the frontoethmoidal suture, anterioposterior growth at the cranial base, and growth at the frontomaxillary and internasal suture [Cohen et al., 1995]. The position of the eyes changes dramatically during development, and insights into the control of orbital spacing come from developmental studies in the chick and mouse [Abzhanov et al., 2007; Brugmann et al., 2010; Cordero et al., 2004]. This work suggests that excess expression of *Fgf8* or *Shh* in the head ectoderm results in broadening of the frontonasal process, thereby leading to abnormally widely spaced eyes. Expression of *Efnb1* in embryonic mutant mice has been shown to be consistent with a role in maintaining separation of neural crest from mesodermally derived tissue in the developing skull vault [Twigg et al., 2004]. Further work will be necessary to assess the effect of altered ephrin-b1 expression on migrating neural crest cells and thereby shed light on the mechanism underlying craniofacial abnormalities in *Efnb1* mutant mice.

## Acknowledgments

We are grateful to J. Hurst, S. Price, and S. Wall for assistance with clinical assessment, to R. Adams for the gift of the mouse lines used in this study, to M. Kowalczyk for advice and assistance, and to C. Healy and P. Sharpe for conducting the micro-CT scanning.
