# Supplementary material for: Duplication of the EFNB1 Gene in Familial Hypertelorism: Imbalance in Ephrin-B1 Expression and Abnormal Phenotypes in Humans and Mice
Source: Hum Mutat. 2011 May 3;32(8):930–8. doi: 10.1002/humu.21521 (PMC3170877; doi:10.1002/humu.21521)
Supplement: Supplementary file 1 [file humu0032-0930-SD1.pdf]

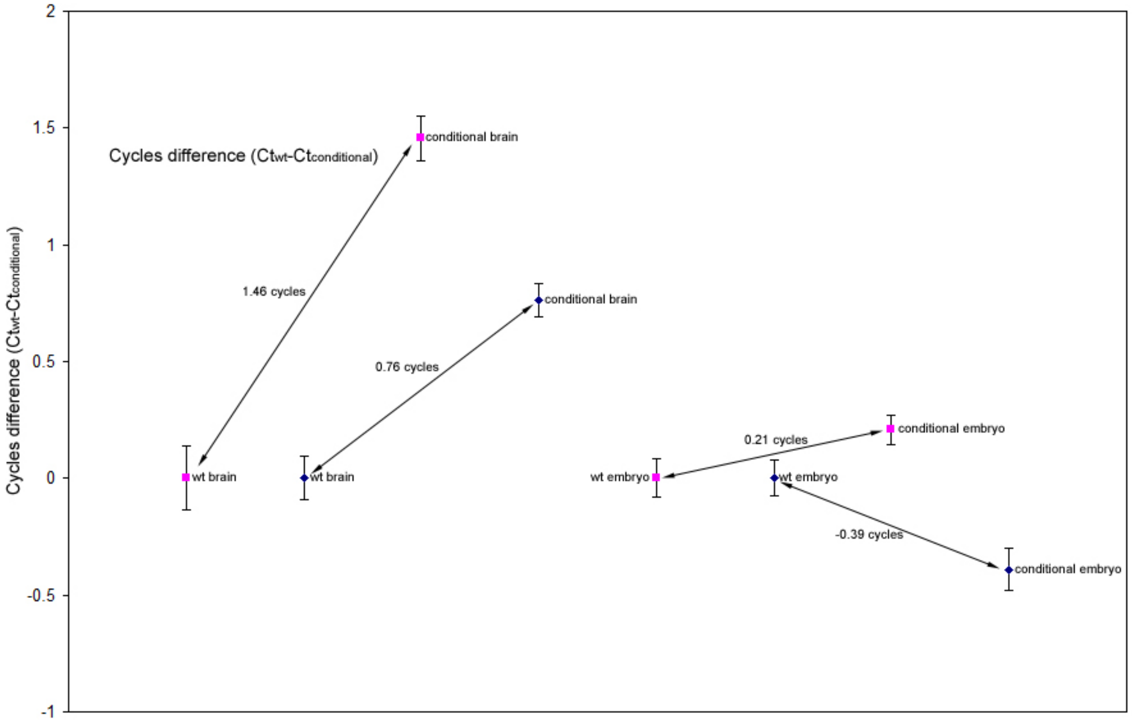

Supp. Figure S1. Expression of wt and conditional *Efnb1*^{Lox} alleles measured by quantitative RT-PCR in tissues isolated from hemizygous male mice. The number of cycles difference between the Ct value of *Efnb1* and the Ct value of the *Gapdh* (blue diamonds) or *Rplp0* (red squares) control genes is shown. The difference between *Efnb1* and each control gene in wt is adjusted to zero. In *Efnb1*^{Lox} brain tissue (left) a cycle difference of between 0.76 and 1.46 was determined, indicating a 1.69-2.75 fold increase in expression from the conditional allele relative to wt. By contrast, in E15.5 embryonic heads the Ct point for the *Efnb1*^{Lox} sample occurred 0.21 cycles earlier (*Rplp0* control) or 0.39 cycles later (*Gapdh* control) than in the wt sample indicating a +1.16 fold or 0.76 fold change in expression. Error bars show the SEM.

Supp. Table S1. Microsatellite Markers used to confirm phase between *EFNB1* and *XIST* polymorphisms

**Marker**	**Location**[a]	**Forward Primer 5'-3'**	**Reverse Primer 5'-3'**
770.69	*EFNB1* -770.1 kb	TTCAATATGGGGGTTTTGTG	6-FAM-CACCCAAGATTGACCCTCTA
DXS1111	*EFNB1* -20.3 kb	AATGACCTTTTTGCCTGGAGAC	6-FAM-TCCCATACCTCACTCAGGCTT
ST5	*EFNB1* intron 1	CTTTCCATCATGACCAAGATTAGTAAGG	6-FAM-CCCCCAACAAAGATTCCTTCATGACC
DXS135	*EFNB1* +20.0 kb	TCAGACACAGGAAGCAGTAG	6-FAM-ACTGAATGGGTTTCTGTCAT
ST10	*EFNB1* + 66 .7 kb	CATGCCTGTGTCCCTGCCTTCTGACCCTCTC	6-FAM-CAGGACTGAATTCCATTTAGTTGGTTCTGGG
DXS8079	*XIST* + 50.0 kb	ATCAATAGCAAACTAGCCGA	6-FAM-TCTTTCAAACCAGGAGCA
DXS8060	*XIST* + 391.4 kb	CACAGCCATGTCCTAGCATA	6-FAM-ACCAAACTTGTTAGTGACCTGA
DXS8092	*XIST* + 1050.3 kb	CACCCTATGGCCTAGC	6-FAM-ACCCAAACTTGCTCAGG
DXS8037	*XIST* + 1050.9 kb	AGGCAAGACATCCATTCC	6-FAM-TGACTTTGAGCGAGCAG
DXS1221	*XIST* + 1194.3 kb	CTTGTGAATTTATTTCAGTTATTG	6-FAM-CCTTAGAAGTGGCCCAG

[a] Where markers are centromeric of the specified gene, locations stated are relative to the start of the transcript shown, in cases where markers are telomeric, their location relative to the end of the transcript is given.
